# Advances in 4D‐printed physiological monitoring sensors

**DOI:** 10.1002/EXP.20210033

**Published:** 2021-12-16

**Authors:** M. A. Parvez Mahmud, Trinny Tat, Xiao Xiao, Partho Adhikary, Jun Chen

**Affiliations:** ^1^ School of Engineering Deakin University Geelong Victoria Australia; ^2^ Department of Bioengineering University of California, Los Angeles Los Angeles California USA; ^3^ Department of Biomedical Engineering, Khulna University of Engineering & Technology Khulna Bangladesh

**Keywords:** 4D printing, personalized healthcare, physiological monitoring

## Abstract

Physiological monitoring sensors have been critical in diagnosing and improving the healthcare industry over the past 30 years, despite various limitations regarding providing differences in signal outputs in response to the changes in the user's body. Four‐dimensional (4D) printing has been established in less than a decade; therefore, it currently offers limited resources and knowledge. Still, the technique paves the way for novel platforms in today's ever‐growing technologies. This innovative paradigm of 4D printing physiological monitoring sensors aspires to provide real‐time and continuous diagnoses. In this perspective, we cover the advancements currently available in the 4D printing industry that has arisen in the last septennium, focusing on the overview of 4D printing, its history, and both wearable and implantable physiological sensing solutions. Finally, we explore the current challenges faced in this field, translational research, and its future prospects. All of these aims highlight key areas of attention that can be applied by future researchers to fully transform 4D printed physiological monitoring sensors into more viable medical products.

## Graphical abstract

This perspective focuses on the overview of 4D printing, its history, and both wearable and implantable physiological sensing solutions. The current challenges faced in this field, translational research, and its future prospects are explored. All of these aims highlight key areas of attention that can be applied by future researchers to fully transform 4D printed physiological monitoring sensors into more viable medical products.

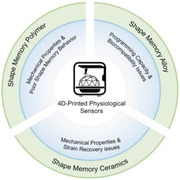

## 1 INTRODUCTION

Technological developments, at their apogee in the twenty‐first century, contribute a large amount of wearable and implantable devices to monitor personal physiological conditions.^[^ ^1^, ^2^, ^3^, ^4^, ^5^, ^6^ ^]^ Three‐dimensional (3D) printing offers versatile architecture of flexible, stretchable, and fine‐surface‐finishing devices at an affordable cost that expands its application from prototype formation to large‐scale production.^[^ ^7^ ^]^ Burgeoning applications are now promising in electronics, robotics, aerospace, healthcare, fashion, and textile.^[^ ^8^, ^9^, ^10^, ^11^, ^12^, ^13^, ^14^, ^15^ ^]^ With the time being, the emergence of smart materials allows 3D printing to leap one step further by adding another time variable, therefore, entering into the four‐dimensional (4D) printing era.^[^ ^16^ ^]^ 4D printing uses 3D printing manufacturing tools and environmental stimuli to change the material's shape and brings a radical transformation in manufacturing.^[^ ^17^ ^]^

Additive manufacturing of 3D and 4D printing with multi‐material availability is found feasible to create parts with varying types and altering compositions within a single layer, ameliorating surface topology and part quality that seem unsurmountable by other means.^[^ ^18^ ^]^ The emergence of advanced materials, such as polymers, composites, hydrogels, ceramics, and alloys, is the consequence of extensive research in additive manufacturing of 3D and 4D printing.^[^ ^19^ ^]^ Numerous progress reports and reviews on that topic have already been published in materials and manufacturing tools aspects.^[^ ^20^, ^21^, ^22^, ^23^, ^24^ ^]^ Nonetheless, a comprehensive summary of 3D and 4D printing on applications in biomedical sectors (implantable and wearable), stability issues of manufactured devices, output accuracy and optimization, and challenges to push them toward commercialization still need a vast amount of research.

In this extensive report, a step‐by‐step illustration of all of these momentous issues will be made. First of all, we will elucidate the background history of additive manufacturing and the emergence of 4D printing in a brief manner. In addition, a comprehensive summary will be made on biomedical applications of 4D printing physiological sensors in both wearable and implantable aspects. Then, we delineate some hitches that impede 4D's massive scale production and bring it into the industrial market with optimal accuracy and lifetime. Finally, we will discuss the current challenges during the manufacturing process and the future outlook of this promising field.

## 2 4D PRINTING

4D printing technology combines stimuli‐responsive materials with a 3D printing process (additive manufacturing), providing a unique and compelling way to create smart materials using only one single step.^[^ ^25^, ^26^ ^]^ 3D printing is described as a process that builds a designed shape using a layer‐to‐layer approach rather than casting or forming from forging and machining techniques.^[^ ^27^ ^]^ Since 3D printing emerged with limitations on producing a single‐printed construct that integrates complex structures, 4D printing came about with the goal to assemble stimuli‐responsive materials such as polymers, hydrogels, alloys, ceramics, and composites. 4D printing demonstrates more advantages over 3D printing: (1) the fabrication process that provides shape changes (2) reduced manufacturing cost and carbon footprint, and (3) manufacturing efficiency.^[^ ^28^ ^]^ The differences between 3D and 4D printing are the two or more stable states offered only by 4D printed structure, in which the structure can shift from one state to another with the presence of stimuli. The additional dimension involves the response of controlled stimuli such as current, voltage, water, heat, electromagnetic radiation, magnetic field, pH, and much more.^[^ ^29^ ^]^ These properties permit 4D devices to integrate a change in shape with the dynamic variation of time. Consequently, the fourth dimension is time.^[^ ^30^ ^]^

The materials in 4D are diverse due to various stimuli acting upon them, including temperature, moisture, electricity, light, current, and magnetic fields. In order to fabricate the design of multiple materials in the structure, mathematical modelling provides inverse problems to find the printing paths. In order to produce 4D printed devices, “smart” or “intelligent” materials are needed. There is no universal definition of what these materials are, but many researchers maintain that they can sense stimuli and produce a useful response. Some examples include piezoelectric, triboelectric, and magneto strictive compounds.^[^ ^14^, ^19^, ^31^, ^32^ ^]^ Consequently, the capabilities of smart materials include shape memory, self‐assembly, self‐actuating, and self‐sensing. Most often, shape memory materials are focused on in 4D printing.

To be more specific, current smart materials utilized in 4D printing include shape memory alloys (SMA), shape memory polymers (SMP), shape memory gels, shape memory ceramics (SMCs), and other shape memory hybrids.^[^ ^33^ ^]^ In this case, SMPs are the most popular in the field. These materials can morph in response to external stimuli and can return to their original shape with the removal of the stimuli. The transformations are simply volume expansions or contraction.^[^ ^34^ ^]^ Therefore, 4D printed materials can be an ideal candidate in the field of sensing.

Self‐sensing materials implement the sensing capabilities to materials that originally do not exhibit this type of property, such as concrete, plastics, textiles, papers, metals, and composites. Specifically, self‐sensing materials provide the designed device with an ability to self‐diagnose and self‐heal,^[^ ^30^ ^]^ which can be turned into physiological sensors.^[^ ^31^, ^35^ ^]^ The currently applicable 4D printing methods include direct inkjet curing, fused position modelling, stereolithography, laser‐assisted bioprinting, and selective laser melting (SLM).^[^ ^36^ ^]^

## 3 TIMELINE OF DEVELOPMENTS

In 1984, 3D printing, also known as additive manufacturing, emerged after Chuck Hull of 3D Systems Corporation filed a patent on a stereolithographic process (SLA).^[^ ^37^ ^]^ This innovative methodology brought about complex shapes that were previously difficult to handle in traditional fabrication techniques. At the moment, 3D printing consists of fused deposition modelling (FDM), stereolithography (SLA), selective laser sintering (SLS), SLM, electron beam melting, inkjet 3D printing (3DP), direct ink writing, and much more. Following its footstep, the idea of 4D printing was introduced in 2012 at a TED conference by Tibbits.^[^ ^38^ ^]^ This marked the beginning of 4D printing, as previously mentioned, where the fourth dimension is time.^[^ ^25^, ^39^ ^]^ With the help of the company Stratasys, Tibbits advanced his printing technique to convert 2D and 1D strands into 3D shapes. Later on, Jerri Qi's research on shape programming of thermally responsive composites became a milestone in the field of 4D printing. They exhibited a shape memory effect when exposed to an appropriate temperature.^[^ ^40^ ^]^

Soon after that, the first research paper on 4D printing was published in 2013. Eventually, the concept of 4D printing was defined as simply 3D printing and time has evolved over the years. Today, 4D printing is defined as the change in shape, property, and functionality of a 3D printed structure over time when exposed to stimuli as mentioned in the previous section.^[^ ^41^ ^]^

## 4 4D PRINTED PHYSIOLOGICAL MONITORING SENSORS

### 4.1 Wearable

In recent years, wearable bioelectronics have been accelerating as tools for healthcare monitoring. Recent advancement in 4D printing techniques has rendered the field with extensive research opportunities.^[^ ^42^, ^43^, ^44^, ^45^ ^]^ Because of the changes induced by external stimuli through time from 4D printing devices, designing and fabricating real‐time wearable physiological monitoring sensors provide great medical benefits. The current wearable sensors will be covered comprehensively in this section.

Recently, Chen *et al*. devised a printed integrated sensor‐actuator (PISA) by applying 4D printing bioinspired microstructure strategy.^[^ ^46^ ^]^ The device offered exciting functionalities such as action/position/posture self‐sensing and active sensing. The PISA could self‐sense temperature and shape change. The main components of PISA's 4D printing materials were carbon black nanoparticles, with features in temperature and pressure resistance, and polylactic acid (PLA), a shape‐memory biodegradable polymer. These materials were linearly filled and printed by an FDM printer.

In 2018, Wang's lab incorporated triboelectric nanogenerator and SMP for self‐power mechanosensation sensors. The SMP‐based triboelectric nanogenerator (STENG) by Liu *et al*. could act as a biosensor and energy harvester.^[^ ^47^ ^]^ Taking into consideration the properties of SMPs and conductive liquid electrodes, the stretchable sensor harnesses biomechanical energy to shape change when activated by a thermal stimulus. The STENG's stretchability, easy adaptation to different surface topographies, and shape‐changing by thermal activation ability make it an ideal candidate as a wrist splint. Consequently, the wrist splint is an effective medical production to treat carpal tunnel syndrome.

Smart materials can also be incorporated as smart textiles. The smart aspect of textiles involves the shape memory materials such as alloys and polymers. The materials can be interwoven into clothing. A commercialized example includes the active sports clothing called Diaplex by Mitsubishi Heavy Industries, using SMP. The Diaplex senses the body temperature and regulates it by keeping down moisture permeability in the cold to keep the body temperature from dropping and vice versa.

Graphene‐based compounds can act as smart materials due to their mechanical, thermal, and electrical properties.^[^ ^48^ ^]^ They are fabricated into strain sensors that can be implemented in healthcare monitoring.^[^ ^49^, ^50^ ^]^ The graphene‐based gas and biomolecule sensors are combined with portable devices for air monitoring and drug detection. Other types include humidity and temperature sensors using graphene materials that act as hydrometers or thermometers. These sensors can be incorporated into wearable devices.

The field of 4D printed wearable physiological monitoring sensors is rather new. Researchers on these devices have allowed for more novel and low‐cost productions due to the abilities offered by the 4D printing industry. Different devices have been proposed to provide smart materials that can support long‐term and real‐time sensing. The exploration of more smart materials and robust designs can provide more comfortable physiological monitoring sensors with improved wearability and versatility.

### 4.2 Implantable

Implantable biosensors offer a wide variety of functions like sensing, monitoring, organization of organs, a tissue or cell generation, proliferation, and stimulation. Although the current development of 4D printing sensors is explored with electronic devices,^[^ ^51^ ^]^ multi‐material origami,^[^ ^52^ ^]^ and composite hydrogel,^[^ ^53^ ^]^ its application is most promising for medical devices because of their autonomous deployment. According to data on 18 January, 2019, 6062 people were listed on the NHS organ transplant waiting list in the United Kingdom.^[^ ^54^ ^]^ 4D printed sensors and devices are able to replace the functioning organ. As a result, they could contribute to alleviating the shortage of organ transplantation for medical care.

**FIGURE 1:**
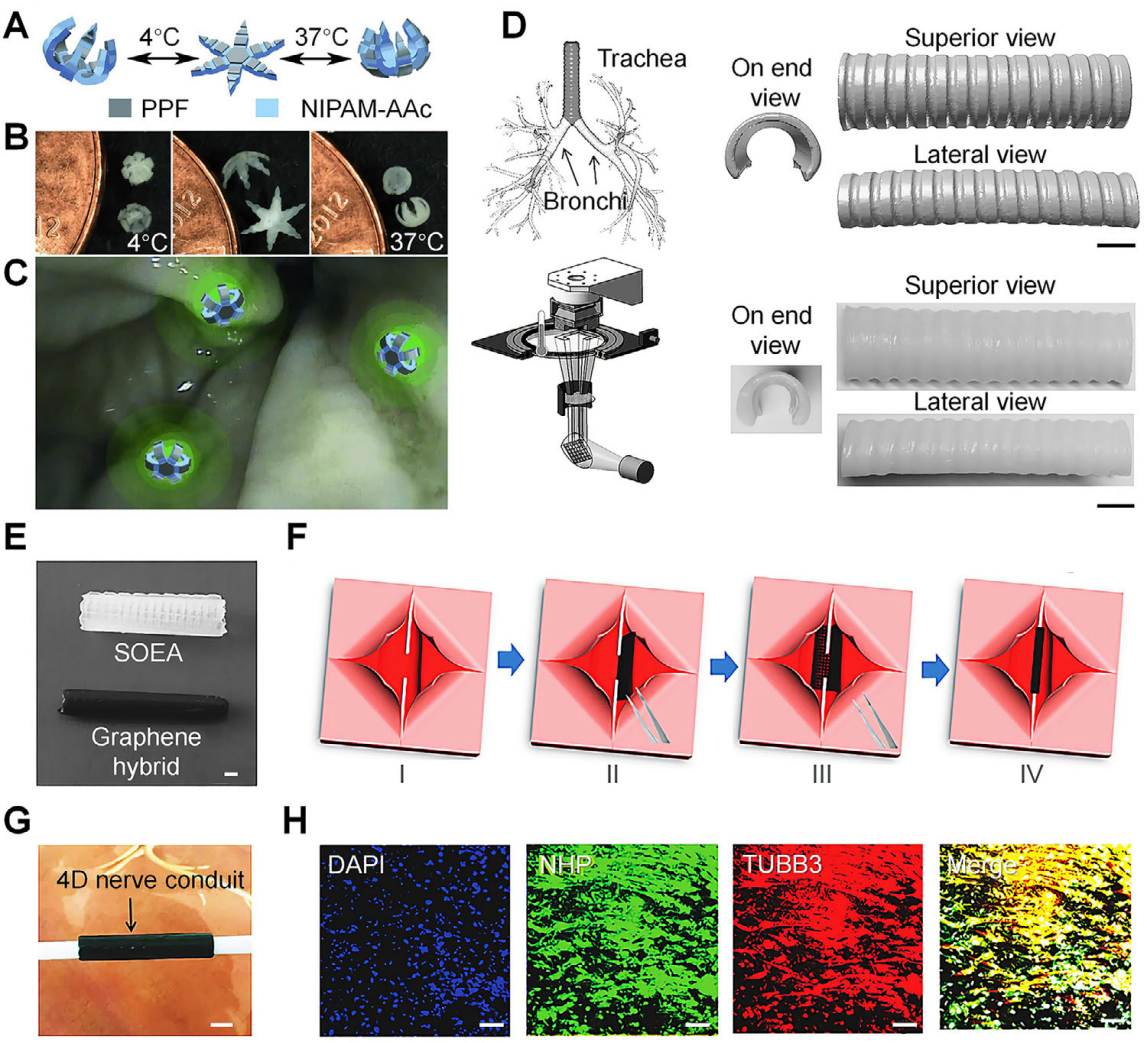
Medical and therapeutic applications of 4D printed devices that possess sensing capability. (A) Rigid PPF panels and thermo‐responsive pNIPAM‐AAc hinges that can close and open within the body temperature limit. (B) Approximate closing temperature 4 °C. Increasing the solution's temperature can close the theragrippers at 37 °C in opposite directions. (C) Colon attachment of the sensor, fluorescent drug release at a targeted site. Reproduced with permission.^[^ ^55^ ^]^ Copyright 2014, John Wiley & Sons. (D) MRI scan support to form a digital model of a tracheobronchial tree. Printed 4D model (right side) of the structure. Scale bar, 1 cm. Reproduced with permission.^[^ ^90^ ^]^ Copyright 2016, John Wiley & Sons. (E) Reprogrammable 4D nerve guidance conduit without (white) and with (black) graphene. Scale bar, 2 mm. (F) Full entubulation by means of thermomechanical programming of the 4D nerve guidance conduit. (i) Damage nerve is shown as two stumps of a severed nerve. (ii) 4D printable nanohybrid construct positioned under damage nerves (black flat part). (iii) Reprogrammable nanohybrid automatically generates its original body structure under body temperature. (iv) The nerve is fully wrapped by the conduit. (G) Photographic illustration of 4D conduit. Scale bar, 2 mm. (H) Neurogenic differentiation of human mesenchymal stem cells (hMSCs) on 4D printed conduit is shown as immunofluorescence images by real‐time PCR technique. Scale bar, 100 μm. Reproduced with permission.^[^ ^79^ ^]^ Copyright 2018, John Wiley & Sons

Figure 1 summarizes some medical and therapeutic applications of 4D printed devices that possess sensing capability and produce corresponding output. Malachowski *et al*. proposed a hydrogel‐based implantable, biodegradable, and photopatternable poly(propylene fumarate) (PPF) and poly(N‐isopropylacrylamide‐co‐acrylic acid) (nNIPAM‐Aac) theragripper (Figure 1A), manufactured using photolithographic 3D printing approach.^[^ ^55^ ^]^ pNIPAM with PPF, showing a modulus three times higher than that of most other hydrogels,^[^ ^56^, ^57^, ^58^ ^]^ was able to form robust gripping sensors (Figure 1B) with sharp tips to secure precise penetration without damaging the tissue. Material composition and sensing properties of the theragrippers allow them to be an ideal delivery vehicle for the targeted site of gastrointestinal tract (Figure 1C) under thermomechanical and chemical sensing. It senses body temperature and can close or open around 4 to 37 °C, which is found auspicious in in vivo sensor applications.

In another work, Zerek *et al*. developed an SMP‐based sensing device using SLA printing method that can be employed in human trachea (Figure 1D). Semi‐crystalline‐methacrylated polycaprolactone (PCL) displays hydrophobic nature and application in tissue engineering, drug delivery, and biomedical devices.^[^ ^59^ ^]^ The 4D airway stent can be thermally actuated and an in vitro experiment was done using a thermal chamber. It was hypothesized that resistive heating by combining this device with TENG could also be possible. But for patient safety and efficacy issues, a ferromagnetic filler phase was required that ensured Curie‐regulated inductive heating, which is still a challenge.^[^ ^60^ ^]^ However, compared to the conventional airway stent, this 4D printed stent is highly promising in future animal models as an implantable device.

Furthermore, in 2018, Miao *et al*., designed and manufactured a programmable 4D nerve conduit to repair peripheral nerve injuries, as shown in Figure 1G. Addition of only 0.8% graphene (black) showed more curvature and flexibility as well as tighter bonding with nerves than without graphene (white) conduit, as shown in Figure 1E. Figure 1F shows a step by step approach of a well‐integrated conduit that provides sufficient tension to the assumed stumps, which are damaged by injury and subsequent axonal regrowth. The thermomechanical programming and thermal sensing enable the desired closed and opened phase in the shape memory device. Graphene significantly increased conduit conductivity and facilitated neural differentiation.^[^ ^61^ ^]^ Figure 1H shows both the neural differentiation and alignment of human mesenchymal stem cells hMSCs. Applications of implantable devices expand rapidly and 4D printing sensors and devices would be one of the most possible fits among the existing technology.

## 5 DISCUSSION

### 5.1 Sensing challenges and future prospective

Some current challenges involve the lack of techniques for smart material production. For example, high‐quality graphene production is expensive, controllable, and scalable at the moment, which paves the path for researchers to improve techniques in the future.^[^ ^49^ ^]^

Another important challenge involving sensing is the biocompatibility and toxicity of the materials, especially if they are implantable. The functionality of smart materials to change shape can have negative effects. Consequently, in the future, it is necessary to investigate the toxicity of these smart materials both in vitro and in vivo.

One key challenge in this field is the ability to visualize and communicate the material properties physically and digitally and can specifically allow the materials to transform at the right time in specific locations.^[^ ^33^ ^]^ Certain designs of materials for 4D printing medical applications require them to adhere to safety requirements, be biocompatible and biodegradable, meet the local tissue‐specific requirements, and exhibit a high magnitude of elasticity for both wearable and implantable sensors.^[^ ^62^ ^]^

### 5.2 Accuracy and stability issues

**FIGURE 2:**
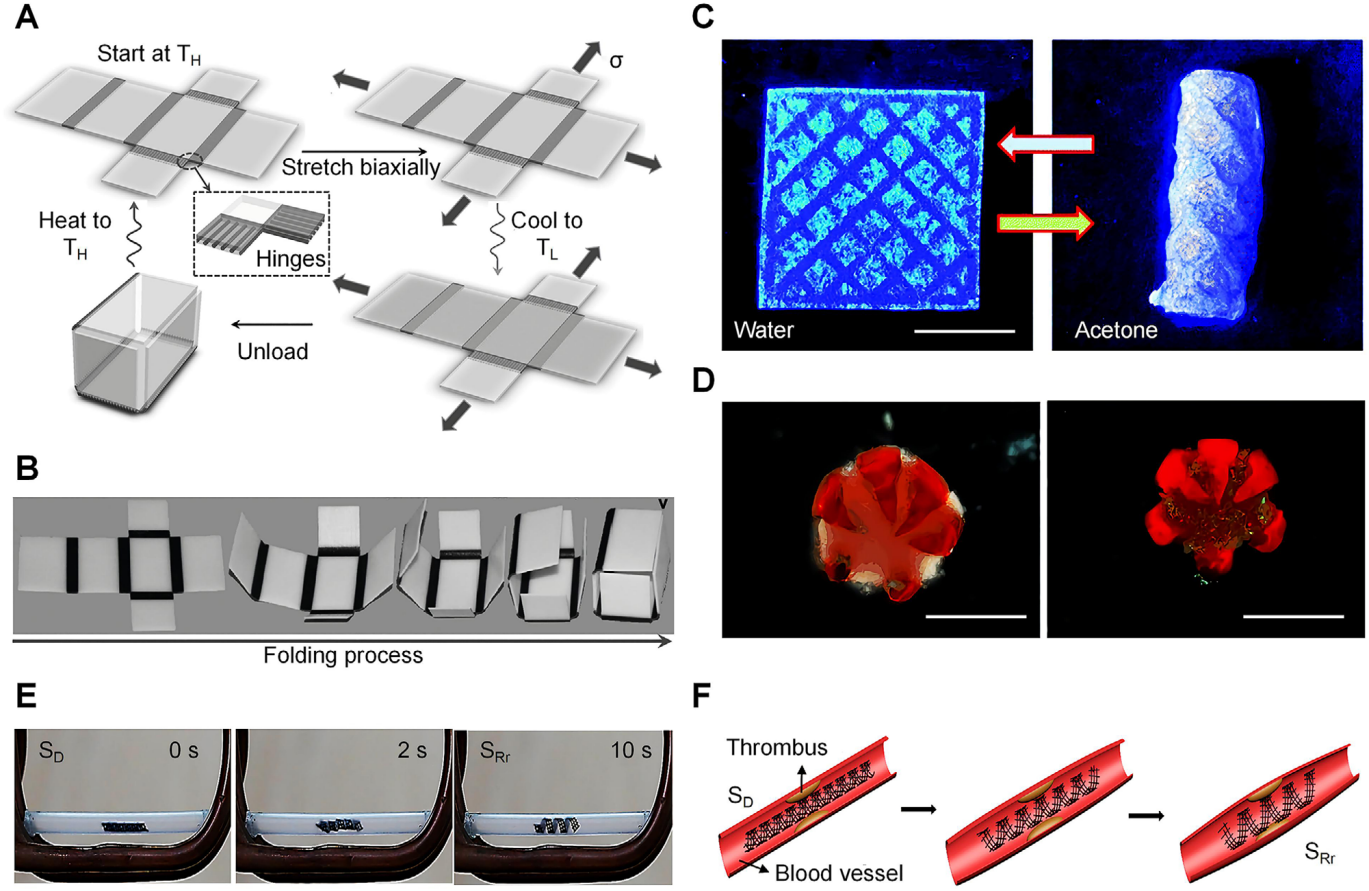
Recent 4D printed materials and their operational performances. (A) Schematic illustration of a thermo‐sensitive materials based self‐folding and opening box. (B) Photographic representation of the folding process. Reproduced with permission.^[^ ^91^ ^]^ Copyright 2013, American Institute of Physics. (C) chemically responsive smart polymer‐based self‐rolling container, actuated by acetone solution. Adapted with permission.^[^ ^92^ ^]^ Copyright 2018, Royal Society of Chemistry. (D) Optical and fluorescent images of the disconnected theragripper compactly locked around and gripping a clump of cells. Scale bars, 1 mm. Adapted with permission.^[^ ^55^ ^]^ Copyright 2014, John Wiley & Sons. (E) Demonstration of a magneto‐responsive restrictive shape recovery process. (F) 4D scaffolds application in intravascular stent. Reproduced with permission.^[^ ^93^ ^]^ Copyright 2016, American Chemical Society

Notable advances in materials science and 3D printing drive researchers to design stimuli‐responsive (e.g., thermal, electric, magnetic, photo, chemical, and humid) materials to enter into the era of 4D printing. Although promising, this field encounters several limitations at its rudimentary stage. The accuracy and stability issue of printed devices are some of them. The accuracy and stability with robustness of 4D printing technology for physical applications largely contingent upon some aspects like flexibility, lightweight, and SMP of materials. Recent advancements in 4D‐printed materials in respect to operational performance and successful applications are shown in Figure 2. The real‐time applications of sensing demonstrate excellent accuracy and stability for these structures.

Multi‐material uses and complex geometry of 3D‐printed structure can certainly increase the performance of the parts with mechanical stability.^[^ ^18^ ^]^ To boost the stability and accuracy of 4D‐printed sensors, researchers focused first on versatile materials availability such as pNIPAM‐AAc soft‐hydrogels, SMA, SMC, SMP, silicone elastomer, and polypyrrole (PPy).^[^ ^36^, ^53^, ^63^, ^64^, ^65^ ^]^ Topological optimization can ensure the highly complex design and the use of composite can target most functional points of a structure to enable stability and ameliorate the existing inconvenience in stability issues.^[^ ^18^ ^]^ Huang *et al*. proposed a promising electro‐hydrodynamic printing technique for 3D printing at micro/nanoscale for various materials.^[^ ^66^ ^]^ SMPs gain substantial attraction to researchers because of their ease of printability and glass transition temperature (Tg) properties. Materials are programmed under specific heat and mechanical treatment above their Tg. By cooling, they are given temporary shape and subsequent heat above the Tg returns the material to its permanent shape. Ge *et al*.^[^ ^67^ ^]^ developed an SMP flower which provided excellent thermo‐responsive behavior and blossomed upon heating above its Tg. In another work, Yoon *et al*. fabricated a self‐folding cubic capsule sensor with high throughput output that could pattern and assemble many structures at once.^[^ ^68^ ^]^ After assembling this architecture, it can be loaded with cargo serving as a drug delivery vehicle. Likewise, Zhang *et al*.^[^ ^46^ ^]^ produced cellulose stearoyl ester‐based thin hydrophobic layer that could react faster with precision upon moisture sensing. Moreover, Okuzaki *et al*.^[^ ^69^ ^]^ developed a PPy film‐based origami micro‐robot that can drive through a humid environment and sense both moisture and electricity. Sequential multi‐stage folding and cross‐folding is the key issue to design complex structures and maintain precise stability at an intricate level. 4D printed devices offer three basic benefits: (1) multi‐material availability, (2) ample 3D printing methods with complex shapes, and (3) variety of response/sensing parameters with perfect accuracy. These kinds of features expand 4D applications in plant morphogenesis,^[^ ^70^ ^]^ medical wearable and implantable devices,^[^ ^71^, ^72^, ^73^, ^74^, ^75^, ^76^ ^]^ and soft robotics.^[^ ^36^ ^]^ Azam *et al*. proposed a container made from biodegradable PCL hinge and photoresist panels with porous structure that can release content upon heat sensing.^[^ ^77^ ^]^ In addition, Malachowski *et al*. fabricated a thermo‐responsive multi‐figured theragripper to serve controlled drugs in the gastro‐intestinal tract upon sensing of body temperature.^[^ ^55^ ^]^ Another successful and accurate treatment of tracheobronchomalacia has been made by Morrison *et al*.^[^ ^78^ ^]^ using 4D‐printed external airway splints sensor. Furthermore, the overall output performance and sensing capability of 4D‐printed devices are satisfactory with stable applicability and accuracy.

### 5.3 System integration issues

**FIGURE 3:**
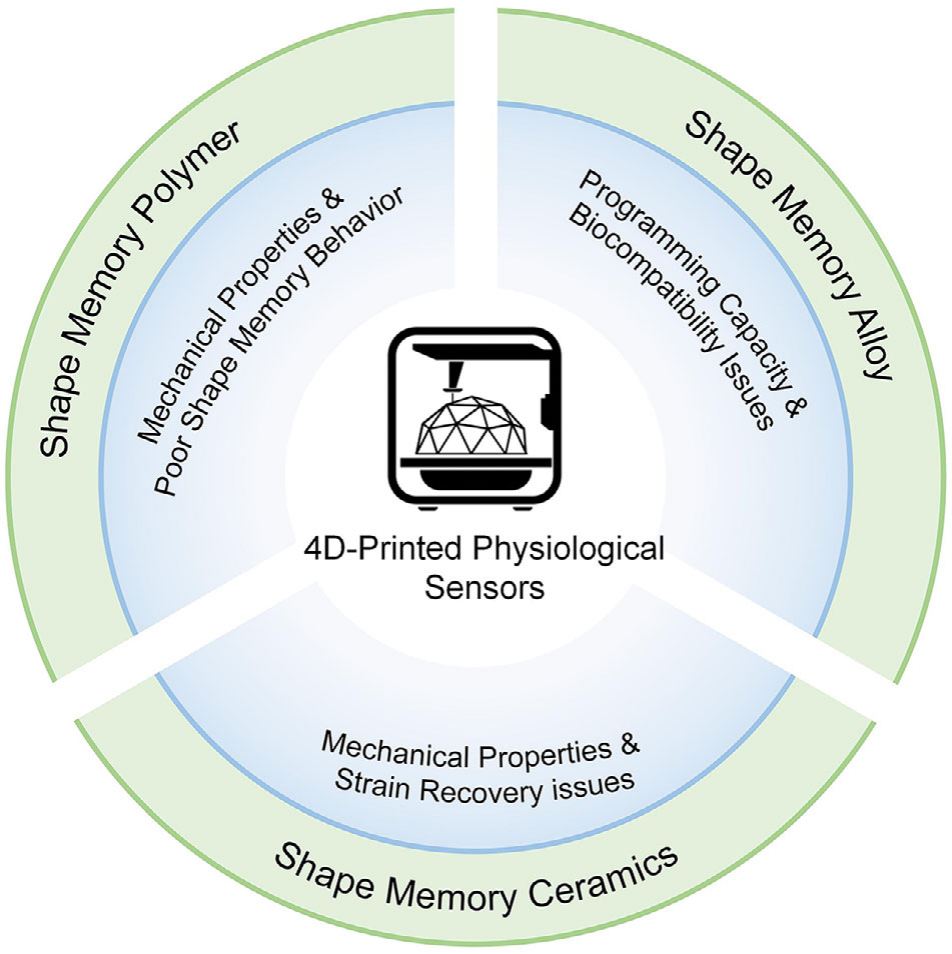
Consideration issues of multi‐materials integration with other systems. SMP: refs. [88, 92, 93]. SMA: refs. [88, 94, 95]. SMC: refs. [36, 95, 96, 97, 98, 99].

4D‐printed structures are compatible to use in a wide range of applications due to their sensing capabilities and easy‐integration abilities. Versatile additive manufacturing methods allow SMMs with high stretchability, deformability, and flexibility that contribute most to their integration with other systems. The present applications of 3D‐printed sensors are investigated in numerous fields, including nerve regeneration,^[^ ^79^ ^]^ proliferation of mesenchymal stem cells,^[^ ^80^ ^]^ soft robotics, self‐changing actuators,^[^ ^67^, ^81^, ^82^, ^83^, ^84^, ^85^ ^]^ multifunctional sensing,^[^ ^69^ ^]^ and self‐healing,^[^ ^86^ ^]^ which indicate their potential of integrations with other devices and systems. Despite these integration compatibilities, 4D‐printed sensors integration at micro/nanoscale yet encountered myriad challenges that must be attained before achieving commercialization. Some integration issues related to their constituent materials are shown in Figure 3. For SMP, the issues focus on: (1) Low density and low tensile strength, (2) degradation and self‐healing capabilities, and (3) low thermal conductivity and shape memory behavior. Also, the development of shape memory metals is trapped by: (1) High tensile strength and moduli, (2) high cost, (3) high density and complicated programming, and (4) biocompatibility and biodegradable issues. Moreover, for the newly developed SMCs, the barriers occur in (1) strain recovery issue and (2) low density and tensile strength issues. These issues can be described by the following key aspects. First is biocompatibility and biodegradability with the biological system. Biological systems are highly immuno‐sensitive and responsive to foreign objects. Biocompatibility of materials encountered frequent hazards to match with multiple bio‐environments. In addition, biological parts are not holistic and cannot be patterned in a particular shape. So, integration and impersonation to the complex structure of the body are always difficult to handle. Finally, device design and material programming to integrate with other systems and optimization of sensing output play a momentous role in integration issues.

### 5.4 Translation to the commercial markets

4D printing with additive manufacturing started its journey with the invention of Skylar Tibbits in 2013, less than a decade ago.^[^ ^38^ ^]^ Since then, a vast amount of research has been conducted to exploit such technology into industrial sectors to commercialize products. Being such a fledgling field, its advancement is still confined to laboratory research rather than commercial market products. To ensure rapid manufacturing into commercial sectors and to achieve a high degree of production accuracy, some crucial challenges must be attained.

Bito *et al*.^[^ ^87^ ^]^ developed some ultra‐low‐cost solutions to produce 3D/4D‐printed sensors at an extensive level. The proposed fabrication technology was able to produce fundamental circuit elements like inductors, capacitors, and antennas, as well as some active components like transistors and diodes, that meet the mass fabrication, high resolution, and flexibility requirements. However, the commercialization of 4D printing devices encountered additional cost considerations while trying to design complex shapes.^[^ ^88^ ^]^ To fix this issue, much more innovative ideas and research are needed in order to develop advanced manufacturing tools with multiple materials availabilities. Rajaraman *et al*.^[^ ^89^ ^]^ proposed a cost‐effective dynamic, stretchable, and packaged 3D printed architecture with shape memory recovery. This biosensor showed an excellent electrical function and returned to its resting state naturally after subjecting to the extreme strain of bending, twisting, or stretching. The SLA process, monolithic structure with high resolution, tunable flexibility, and biomaterial uses allowed the sensor to be used for lab‐on‐a‐chip application at a commercial level.

Another important issue lies in 4D printing's functionality and maximizing output performance. Proper selection of impeccably matched materials is difficult, thus jeopardizing the output optimization and impeding manufacturing products. Rafiee *et al*.^[^ ^23^ ^]^ discussed in detail about the evaluation and proposed one probable solution for production efficiency for 4D printing devices. Some other issues that play crucial roles in manufacturing products that need to be considered are: (1) mechanical properties such as compression and tension that cause decrease interfacial bonding between printed layers; (2) fatigue and slow mechanical response of the manufactured materials; (3) pre‐determined program‐based actuation and motion; (4) potential dimensional accuracy and reversibility of materials structure; (5) compatibility with intricate shape and environment of the human body. Finally, it is anticipated that current hitches in this novice but cutting‐edge technology will come to an abrupt end very soon. The extensive research in 4D sensors and advances in the manufacturing process will bring commercially fit devices in the near future.

## 6 CONCLUSIONS AND OUTLOOK

Additive manufacturing with 3D‐printing combined with time variable to develop 4D‐printed devices has expanded its application to diverse fields. Compared with present 3D‐printing systems, 4D‐printing technology boosts the self‐active sensing capacity and programming capacity of their products. As mentioned in the formal passages, 4D‐printed devices not only share the advantages of flexible designing, fine‐surface finishing, and economic expense with 3D‐printed ones, but also play an irreplaceable role in the implantable and wearable system due to the thermal/mechanical sensing capacity that enables delicate control with desired closed and open phase. In this progress report, recent advances in 4D‐printed sensors and their potential uses are illustrated respectively in biomedical, sensing, and energy harvesting capabilities. Key challenges in wearable and implantable issues, output stability, and commercialization of products with optimal output are also discussed in a meticulous way.

This field opens a new horizon of prototype formation and microscale manufacturing that could not be possible by other means. The availability of protean materials has given another dimension to reach its pinnacle. The impeccable combination of 3D printed technology and advanced materials in 4D sensors envisioned its future application in implantable and wearable bioelectronics, defence, soft robotics, fashion, automobiles, smart actuators, aerospace, and renewable energy sources. To exploit these devices for the betterment of human civilization and produce reliable devices, the following challenges should be overcome. First of all, printing technology like inkjet/poly‐jet, SLA, and FDM are the most common forms of printing techniques for prototype formation. These modelling techniques are quite suitable for nonmetal elements. The availability of fully developed 4D printers with multi‐material printing is still challenging. In addition, the development of smart materials is a prerequisite for the foreseeable advancement in 4D printing. Existing research on 4D‐printed soft robots using mainly SMMs exhibits a limited number of reverse actuation. To solve this issue, much more research is needed on thermo‐mechanically programmed materials that can provide the liberty of reversible actuation. It is anticipated that the successful development of 4D materials will reduce the need for costly replacement and repair for implantable devices. Moreover, external stimuli response under continuous loading or extreme environment should be considered for a particular device. Likewise, the complex shape of the body impedes the integration and designing process. Similarly, variant in vivo environments and immuno‐sensitive nature of the body behave harshly to foreign objects like 4D implantable biosensors. These kinds of inconvenience force researchers to develop devices in a biocompatible and biodegradable manner.

Finally, it is hopeful that 4D printing devices will probe new dimensions and lead to a paradigm shift in every aspect of human life in the near future. Although in its nascent stage, more advanced tools will be developed in the near future that can provide highly intricate structures with smart operation. Smart materials with biocompatibility and biodegradability are probed to extend their operation in nerve and tissue generation, cell proliferation, and scaffold for the body. For the sectors like energy harvesting and sensing, it will be the most classical field of improvement among the existing technologies.

## CONFLICT OF INTEREST

The authors declare no competing financial interest.
